# Development of New Lignin-Based Coatings with Ultraviolet Resistance for Biobased Composite Materials

**DOI:** 10.3390/polym16152175

**Published:** 2024-07-30

**Authors:** Patricia Ares-Elejoste, Ana Pérez, Koldo Gondra, Rubén Seoane-Rivero

**Affiliations:** GAIKER Technology Centre, Basque Research and Technology Alliance (BRTA), Parque Tecnológico de Bizkaia, Edificio 202, 48170 Zamudio, Spain

**Keywords:** biobased composites, lignin, UV resistance, flax fibers, sustainability

## Abstract

Nowadays, there is a challenge in searching for more sustainable alternatives to decrease the environmental impact of composite materials. In this work, we fabricate new composites based on a biobased-content epoxy system, lignin, and flax fiber; considering these materials could be promising due to their high renewable content of around 40%. In addition, another key requirement for composites, besides being sustainable, is that they present improved properties such as UV resistance. Therefore, throughout this work, priority was given to improving UV resistance in addition to taking into account sustainability. In order to carry out a complete characterization of the materials developed, the mechanical properties, brightness, and thermal, rheological, and fire behavior of these kinds of materials were analyzed by using vacuum-assisted resin infusion processes. By way of conclusion, it should be noted that the manufactured composite with the optimized formulation showed improved UV resistance using lignin and that it could be applied on internal and external walls according to the railway fire regulations.

## 1. Introduction

The rapid growth of industries and the high demand from customers in different sectors have promoted the need to improve materials in terms of strength, stability, lightness, and cost, taking into account, especially in recent years, the sustainability factor [1,2,3,4,5,6]. In response to this demand, composite materials have been a considerable improvement in the market, since they possess good properties and, in addition, cause less impact on the environment [7,8,9,10,11]. 

Materials formed by two or more components (natural or artificial) are known as composite materials or composites and stand out for having better properties than the components themselves separately. They are designed to provide more lightness, corrosion resistance, durability, and/or efficiency. With respect to the configuration of these types of materials, the vast majority of them are formed by two elements: a matrix and a reinforcing agent, which is adhered to the first one. In summary, it can be concluded that the fiber provides stiffness and strength while the matrix is flexible and not very strong. Therefore, the matrix protects the fibers, gives them shape, and transmits the stresses between fibers [12,13]. 

This growing interest in composite materials is the result of the need to lighten structures, for example, intended for the transportation sector, thus reducing fuel consumption and, therefore, greenhouse gas emissions responsible, to a large extent, for environmental pollution. Over time, this objective has led to an extraordinary increase in the production and diffusion of these types of materials in all industrial sectors. In short, the use of composite materials based on renewable materials (biobased-content resins) obtained from agriculture or biomass is booming. These new materials help to offset the use of fossil fuels and reduce greenhouse gas emissions. Furthermore, in several sectors, in recent years, more sustainable reinforcements have also started to be used. However, it should be noted that natural fibers have certain drawbacks, such as excessive water absorption or low temperature resistance [14,15], which means that they are not always the first choice. Nevertheless, these drawbacks must be solved to promote the use of more sustainable materials and achieve properties similar to those of traditionally used composites [16].

In addition to more sustainable resin systems and natural fibers, the use of renewable additives in composite materials has been considered in recent years in order to improve properties and increase the biobased content.

Since the creation of the pulp and paper industries at the end of the 19th century, a large amount of residual lignin has been incinerated to recover chemicals and produce energy [17,18]. These recovery processes only focused on the energetic power of lignin, but, in recent years, it has become an additive of renewable origin of great interest for producing biobased materials, such as coatings, reinforcing agents, and biofuels [19,20] Lignin is a natural macromolecule found in the cell wall of plants as a result of condensation reactions of different radicals. The presence of different functional groups makes lignin a complex substance; some of the radicals that form lignin are hydroxyl (-OH), methoxyl (CH_3_-O), carbonyl (-C=O-), carboxyl (-COOH), and benzene (C_6_H_6_) [21,22,23,24]. The Kraft process is the primary method for producing lignin from lignocellulosic biomass, and it is important to note that this type of lignin is primarily sourced from the pulp and paper industries as a by-product. In this process, wood is exposed to a water-based solution of sodium hydroxide (NaOH) and sodium hydrosulfide (NaHS) to extract lignin from cellulose [25,26]. In contrast, organosolv lignin is acquired through the use of delignifying solvents as a different approach to pulping technology. Similar to lignosulfonates and KL, the structure of organosolv lignin also contains carbonyl and phenol hydroxyl groups [27].

**Figure 1:**
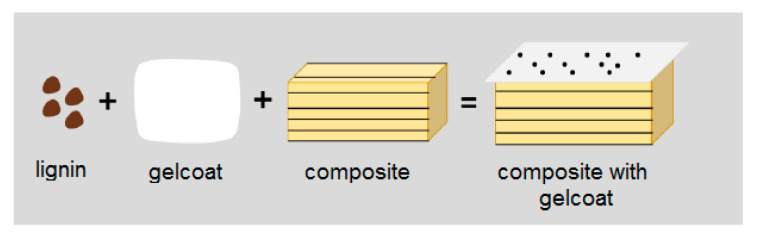
Scheme of the components used for the development of biobased composites with a lignin-content coating.

Throughout this work, we have worked with a biobased-content epoxy resin. These types of resins are presented as viscous liquids that cure when a hardener is added [28,29,30,31]. Although its price in comparison to other resins is considerably higher because of its novelty and renewable raw materials used for its synthesis, it should be noted that they show very good properties compared to other resins such as polyester resins. They have a high adhesion capacity to most surfaces, which ensures good resistance. As far as flammability is concerned, being less flammable than other resins, they are safer and more tenacious because of their greater elongation capacity [32]. As reinforcement, we have worked with flax fiber, within the natural fibers, which is a fiber with high resistance. The structure of these fibers is very complex as they are constituted by a series of elementary fibers in the form of superimposed polyhedrons. These are held together by an interface consisting mainly of hemicellulose and pectin. The typical diameter of these elementary flax fibers is around 10–15 μm, and, on the other hand, that of technical flax fibers varies between 35 and 150 μm [33,34,35]. In this work, the composites were not developed exclusively using a matrix and a fiber. A coating/gel-coat was added to these two elements to obtain better composite surface behavior. Before applying this coating or gel-coat to the composite, different types of lignin were added in different amounts to study the effect that these additives have on the coating and whether or not they improve some properties, such as UV resistance. Figure 1 shows a complete scheme of the composites created and characterized.

In addition to evaluating the UV resistance, throughout this work, other properties such as, for example, fire resistance were characterized as it is of interest to know whether these materials can be used in sectors such as transport. To this end, the cone calorimeter test has been carried out using the standard applicable to the railway sector [36] (UNE-EN 45545 2:2021), as this test is a good method for screening under considerably extreme fire conditions. It should be considered that the composites developed in this work are expected to have a MARHE (parameter used to classify materials in the railway sector) of 90 kW/m^2^ or less in order to be applied to, for example, train interiors and exteriors.

## 2. Materials and Methods

### 2.1. Materials

A commercial epoxy resin system with its respective hardener has been used in this work: Resoltech 1800 + Hardener 1805 ECO. This is an epoxy system from RESOLTECH suitable for resin infusion and injection processes. This system has a resin/hardener ratio of 100:17 with a biobased content of around 36%. One of its most outstanding characteristics is its low viscosity and high mechanical properties with a glass transition temperature of 102 °C. The flax fiber used is a Biotex Flax of 245 g/m^2^ unidirectional weight. Two types of commercial lignins have been studied together with a biobased-content gel-coat (51% bio-content) in order to evaluate the UV resistance of the bio-composites. One of the lignins used is Sigma-Aldrich’s Kraft Lignin (St. Louis, MO, USA), and the other one is Chemical Point’s Organosolv Lignin (Oberhaching, Germany). All materials were used under the manufacturer’s recommendations.

### 2.2. Characterizations

In order to evaluate the reactivity of the system and thus obtain the temperature of the exothermic peak and the enthalpy of cure, the formulations were characterized by differential scanning calorimetry (DSC). For this purpose, a dynamic temperature sweep was performed from 25 °C to 280 °C at a rate of 10 °C/min in a N_2_ atmosphere (50 mL/min) using a 40 μL aluminum pan. Thermogravimetric analysis (TGA) was also performed, which shows the degradation and mass loss of the sample against an increase in temperature. For this purpose, a certain amount of lignin or gel-coat was introduced into the ceramic pan, and this sample was heated from 25 to 600 °C with a temperature sweep of 20 °C/min under an air atmosphere at a gas flow rate of 50 mL/min.

For viscosity measurement, an Anton Paar rheometer, model MCR 501, was used. To carry out the test, a 25 mm diameter plate–plate measuring system was used, and a gap of 1 mm between plates was established. With respect to the test methodology, the following has been established:

- Type of test: rotational test.
- Test temperature: 25 °C.
- Shear rate: (10 s^−1^–100 s^−1^) and (20 s^−1^–200 s^−1^) depending on the system to be measured.

To perform this test, 10 gr samples were prepared. Once the sample was prepared, using a pipette, the sample was introduced into the equipment (on the lower measuring plate), and the viscosity was determined. 

For a correct evaluation of the influence of the resin systems, the different lignins in the composites developed, and the compatibility between the matrix and the fiber, mechanical tests were carried out; the flexural behavior of the specimens was studied at three points. These tests were carried out under the “UNE EN ISO 14125” standard [37]. The fire behavior of the manufactured composites was evaluated by the calorimetric cone. These tests were carried out in accordance with ISO 5660-1:2015 + AMD:2019 [38], classifying the materials according to EN 45545 2:2021 [36]. The FTT cone calorimeter used is Siemens equipment, equipped with an O_2_/CO_2_/CO_2_/CO Ultramat/Oxymat 6 analyzer. The main purpose of this equipment is to quantify the amount of heat emitted by the sample when exposed to controlled levels of radiation. 

The fire behavior was analyzed using several parameters: MARHE (the maximum average heat release rate), THR 1200s (the total heat release after 1200s), Qmax (the maximum heat), and TMLR 1200s (the mass loss rate after 1200s). MARHE is the parameter used to certify materials in the railway sector.

The operating condition used was a radiation power of 50 kW/m^2^ (reaching a specimen surface temperature of 600 °C), with a distance of 60 mm from the specimen to the cone. 

Through the QUV aging chamber, the accelerated aging of materials is analyzed by reproducing the damage caused by rain, sunlight, and dew. In other words, it is used to subject samples to ultraviolet (UV) light and condensation cycles at a controlled temperature. In this case, the fabricated samples were exposed to a total of 500 h of exposure according to the artificial aging method with UVA-340 lamps and condensation effect. For characterizing the aging effect, the equipment used to measure gloss was a BYK Micro Tri GLOSS glossmeter (20°/60°/85°), according to the UNE ISO 2813 standard [39]. Regarding the methodology of use, it is placed on the sample, and a total of 10 measurements are made to obtain a real average value of the entire sample. Color characterization was also performed on each of the samples; the CIELAB method was used to obtain information on the surface color. For color measurements, a Konica Minolta CM-2300d spectrophotometer was used, with the following characteristics:

- Diffuse measurement geometry: reflectance d/8 (8° detection angle).
- Standard observer: 10°.
- Standard illuminant: D65.
- SCI (specular component included).

### 2.3. Manufacturing of Biobased Composites

In this work, the resin infusion process was used to manufacture the target biobased composites. Table 1 shows the composition of the different composites manufactured. To carry out this process, the following steps must be carried out:

1. Preparation of the mold: on a glass plate, Teflon is placed, and release wax is applied to facilitate the subsequent demolding of the composite after the infusion process. 

2. Next, the area on which the composite is to be fabricated is drawn, and the gel-coat is applied and left to cure until it is ‘tacky’. 

3. Thereafter, the stacked flax fibers are placed, with a peel-ply to facilitate the extraction of the plate; a distribution mesh that covers up to half of the fibers to facilitate impregnation; and two tubes, one through which the resin is injected and the other on the opposite side through which the vacuum is created. Once the equipment is assembled, mastic is placed on the edges of the glass and sealed with a film as a counter-mold.

4. Creation of the vacuum: Once the entire glass is covered with the film, one of the tubes is connected to the vacuum pump, and the vacuum is created to eliminate the air inside and favor the compacting of the fibers. It should be noted that when flax fiber is used, the infusion system must be heated in an oven at 50 °C for at least 30 min to eliminate any moisture that may have been absorbed by the fiber.

5. Resin infusion: To proceed with the infusion, the resin and hardener formulation is prepared, and the remaining tube is introduced into the mixture while continuing to create a vacuum. In this way, the resin is absorbed and runs through the dry fiber until it is completely impregnated.

6. Control and monitoring: during the infusion process, the distribution of the resin is controlled to ensure that it reaches all of the necessary areas evenly and that there are no leaks.

7. Completion of the process: once the resin system has been properly impregnated through all of the fibers, the resin inlet is blocked, and the system is left to cure in an oven while maintaining the vacuum at the curing conditions set by the manufacturer.

**Figure 2:**
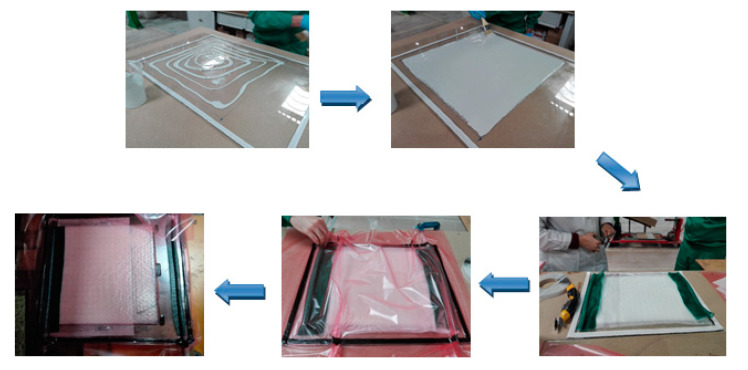
Scheme of composite manufacturing based on infusion process (steps 1–5).

According to the steps aforementioned, in Figure 2, a scheme of some steps of the infusion process carried out can be observed:

As mentioned above, the formulations processed by the infusion process, where different percentages of lignin in the gel-coat have been used (as mentioned in Section 2.1), are listed in Table 1 below. It should be noted that lignin has been introduced in the gel-coat because it will be on the outer face of the composite, and the aim is to improve the UV resistance.

## 3. Results

### 3.1. Thermal Analysis

**Figure 3:**
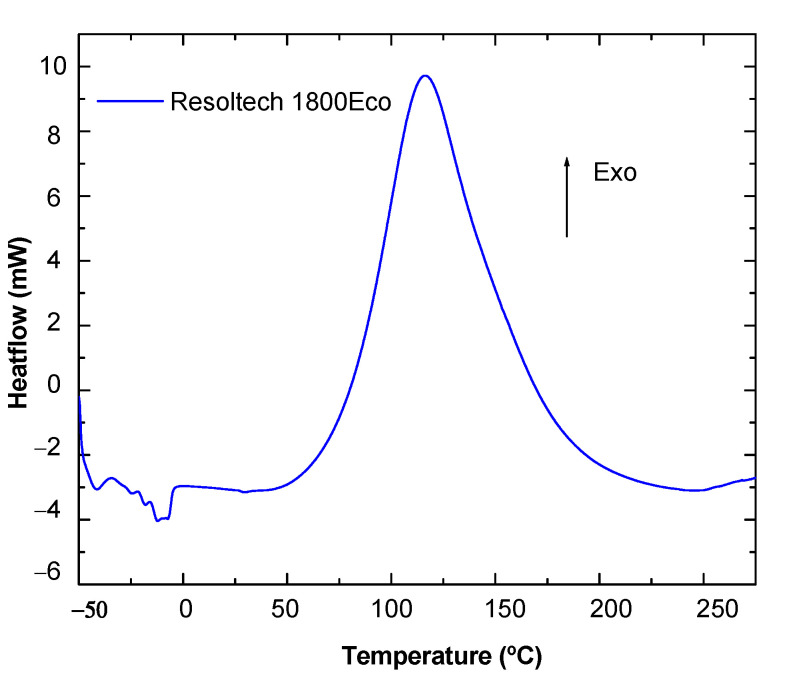
DSC curing results of the system, Resoltech 1800Eco, with the hardener, 1805Eco.

To observe the thermal behavior of the composite, firstly, the resin system used (Resoltech 1800eco, Rousset, Framce) was analyzed by DSC. As shown in the curve obtained (Figure 3), the reaction of the system is exothermic, which results in a heat release due to the crosslinking (curing) reaction.

**Figure 4:**
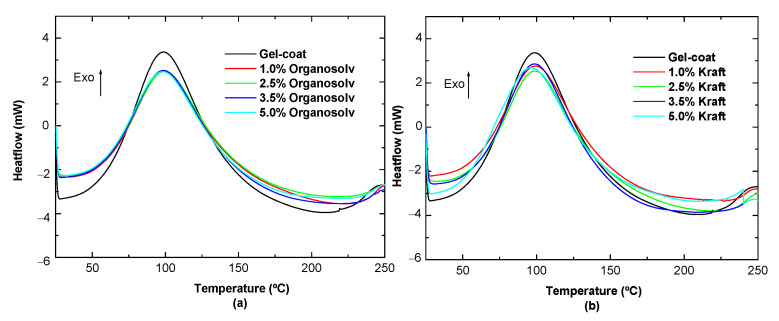
DSC results of the gel-coat with (**a**) Organosolv lignin and (**b**) Kraft lignin.

Furthermore, in Table 2, the data for exothermic heat and maximum exotherm temperature are detailed:

In order to test whether the incorporation of lignin in the gel-coat modifies the thermal properties of the resin system, a study of the curing of different formulations was carried out. In Figure 4, no significant alterations are observed in the curing curves. However, it should be noted that it can be seen that the curing curves of the formulations with different amounts of Kraft lignin do not show significant differences compared to the reference. They are similar to the reference formulation. After the analysis of the curing curves, the theoretical enthalpy of each formulation was calculated to observe if there is an inhibition in the curing of the system. It should be noted that these enthalpy values were calculated from the experimental enthalpy obtained from the software of the differential scanning calorimetry equipment.

As mentioned above, in addition to analyzing the DSC curves, the theoretical enthalpies were calculated in order to confirm or deny if lignin inhibits the curing reaction of the gel-coat. For this purpose, the following equation has been used:

ΔH theoretical = ΔH experimental × (% gel-coat)(1)

Table 3 summarizes the results obtained:

Analyzing the results obtained in terms of theoretical enthalpies, they are somewhat lower than the experimental reference enthalpy (formulation without lignin). However, it should be noted that these differences are not considered significant. Comparing the results with the different proportions of lignin, it is observed that as more lignin is added, it interferes very slightly with the inhibition of curing. In fact, this behavior is similar for both types of lignin, although the experimental enthalpy values for the Organosolv formulations are slightly lower. In conclusion, in general, this type of additive does not interfere negatively with the crosslinking reaction, preventing the full cure of the system.

In order to determine the degradation temperature of the gel-coat used, it was subjected to thermogravimetric (TGA) analysis. This temperature was obtained by establishing tangents manually at the point where the mass percentage suffers a considerable decline. This analysis shows the % mass loss of the sample with increasing temperature. 

**Figure 5:**
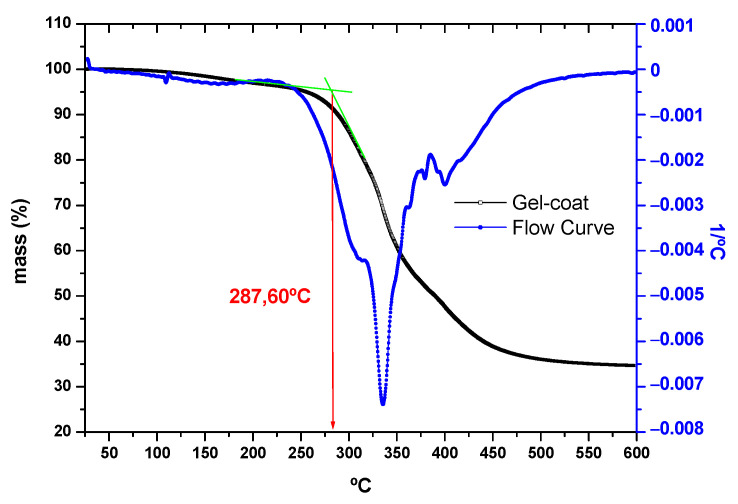
TGA results of the bio-content gel-coat used.

As shown in Figure 5, according to the TGA results of the bio-content gel-coat, the degradation temperature of the gel-coat is 287.60 °C, which shows that it is resistant to relatively high temperatures.

**Figure 6:**
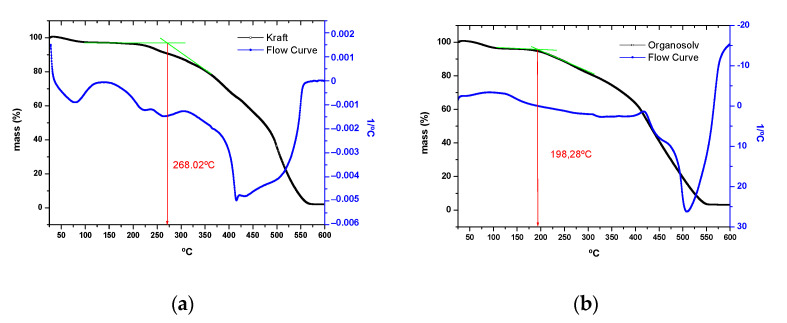
TGA results for the gel-coat combined with (**a**) Kraft lignin and (**b**) Organosolv lignin.

Concerning the lignins used, although both types of lignin are mainly made up of cellulose, they have different degradation temperatures (see Figure 6 for TGA results for the gel-coat combined with (a) Kraft lignin and (b) Organosolv lignin). This temperature, as in the previous case, was obtained by making the tangent of the curve obtained from the percentage of mass lost with temperature (as performed for the abovementioned TGA results of the bio-content gel-coat shown in Figure 5). 

It was possible to verify that Kraft lignin is more resistant to temperature, as its degradation temperature is 268.02 °C. However, Organosolv starts to degrade at 198 °C. It is less resistant to high temperatures. As a hypothesis, since kraft lignin is obtained through chemical dissolution at high temperatures and pressures, it is possible that this lignin has created a higher surface resistance at a high temperature.

Lastly, regarding the mass loss, it can be observed that both Organosolv and Kraft lignins lose much more mass than the gel-coat as the test proceeds. According to the data provided by the software, the following mass percentages are lost depending on the component:

- Gel-coat mass loss: 65%.
- Kraft lignin mass loss: 95%.
- Organosolv lignin mass loss: 93%.

### 3.2. Rheological Analysis 

Prior to the development of the composites with the aforementioned formulations, the rheology (viscosity) of the biobased-content epoxy resin system and the gel-coat (38% bio-based) was studied. Before carrying out the test, both samples were prepared according to the supplier’s recommendations and mixed until a homogeneous mixture was obtained. Once the mixture was prepared, the shearing rate was fixed according to the according to the viscosity shown by the system (visual observation of the mixture).

The results obtained from this analysis showed a viscosity of about 435mPas for epoxy resin, for which a shear rate of 20–200 s^−1^ was used. 

With respect to the gel-coat, this has been analyzed using a shear rate of 10–100 s^−1^ due to the viscosity of the system (higher in comparison to the epoxy system), which was about 12,000 mPas. 

**Figure 7:**
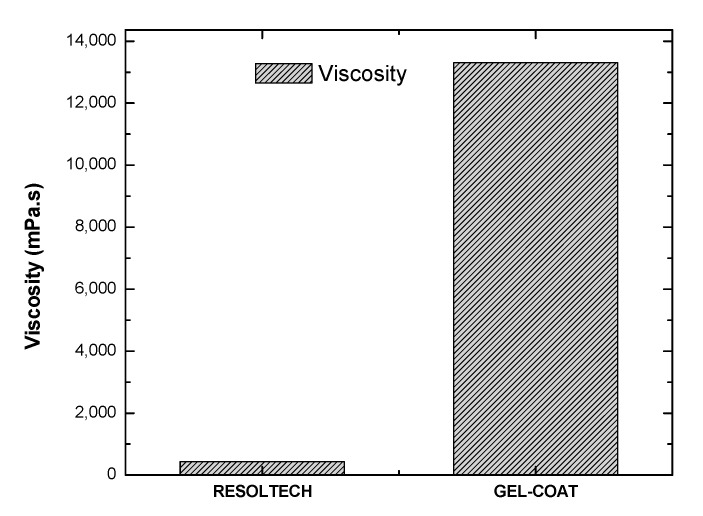
Viscosity of the biobased-content epoxy system and gel-coat.

Both viscosities can be observed in Figure 7:

It can be said that this viscosity was suitable for lignin dispersion, as lower viscosities could lead to lignin precipitation in the mixture. Finally, it was not possible to measure the gel-coat formulations with the lignins together because of their high viscosity. 

### 3.3. Mechanical Characterization

Once the composites were manufactured, their mechanical behavior was studied. It should be noted that before testing the composite coupons, the specimens were checked in case there might be significant differences in thickness or delaminations after processing. These coupons were tested under the standard detailed in Section 2.2.

**Figure 8:**
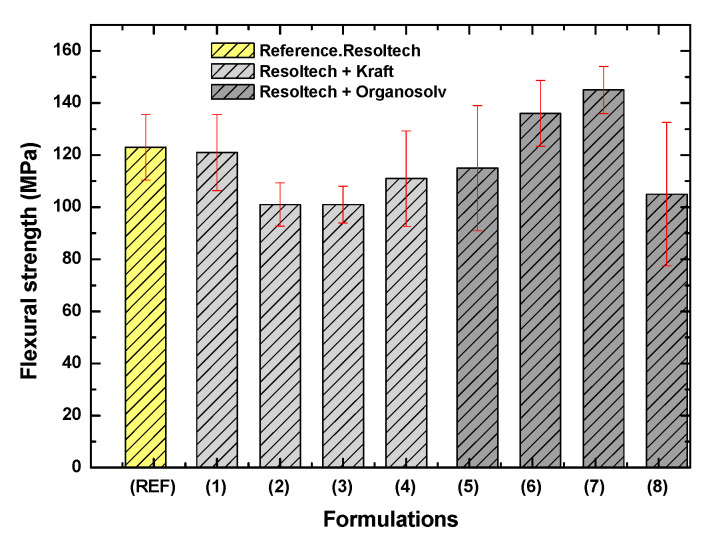
Flexural strength of the developed biobased composites.

With respect to the composites developed, it can be seen that 1%wt lignin, both Kraft and Organosolv, does not significantly influence the strength values when compared to the reference value. However, as more lignin is added, in the case of Kraft lignin, a decrease in mechanical strength is observed. On the other hand, in the case of Organosolv lignin, an increase in flexural strength is observed as more lignin is added. It should be noted that with 5%wt Organosolv lignin, the properties are decreased, possibly due to a saturation of the gel-coat. Formulations 6 and 7 (formulations with organosolv lignin) show an improvement of around 20% in terms of strength, reaching 140–150 MPa (Figure 8). 

The improvement seen in the flexural strength with the organosolv lignin might be because, as this lignin is obtained by less aggressive technology than kraft lignin, which is obtained by precipitation in an aqueous medium, it is likely to be of higher quality, its structure is more intact, and it may create a crosslinking reaction with the gel-coat. 

**Figure 9:**
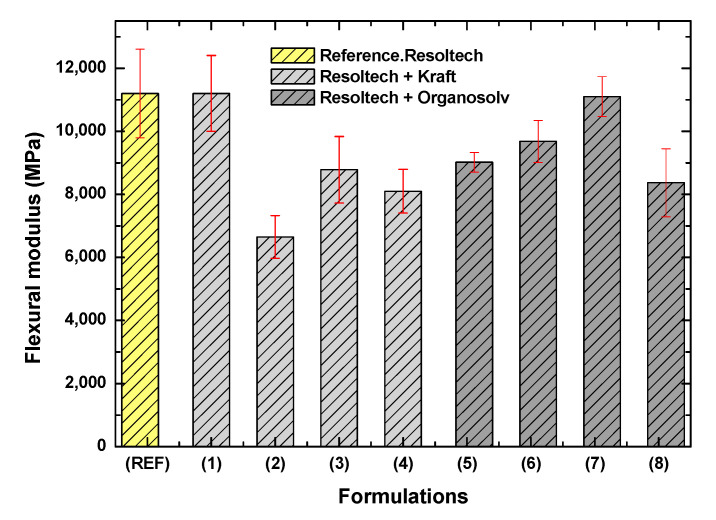
Flexural modulus of the developed biobased composites.

Regarding the flexural modulus, Figure 9 shows the results with the standard deviations of the composites developed. As can be observed, the addition of lignin decreases the flexural modulus, except in formulations 1 (1 wt Kraft lignin) and 7 (3.5%wt organosolv lignin), where the modulus is similar to that obtained in the reference formulation. With regard to formulation 2, which shows the lowest flexural modulus, it is believed that there may not be good dispersion of the lignin in the gel-coat. Specifically, the highest modulus achieved is around 11,000 MPa, which is reached in the following formulations:

- Reference.
- Formulation 1 (Resoltech + 1%wt Kraft lignin).
- Formulation 7 (Resoltech + 3.5%wt Organosolv lignin).

### 3.4. Color and Gloss Characterization

**Figure 10:**
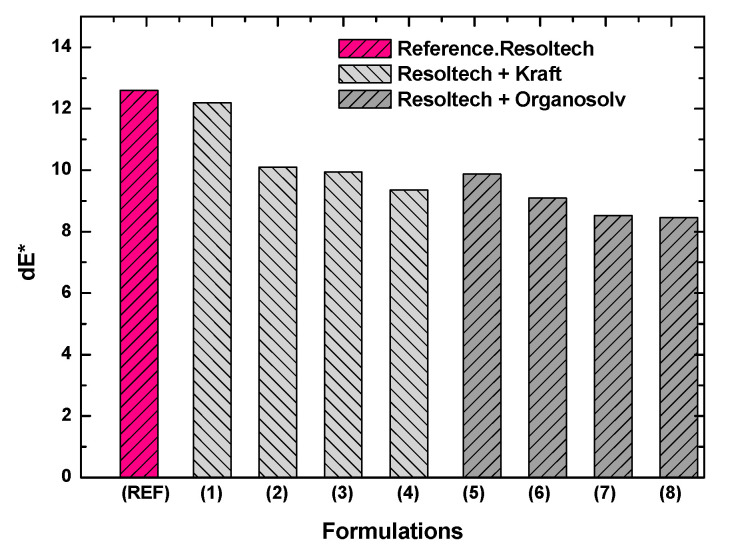
Color change after QUV aging of the developed composites.

In order to study the UV resistance of the gel-coat, which is the aim of this work, the samples were subjected to a QUV aging process. Before and after the test, the color of the surface was measured according to the CIELAB scale in order to calculate the change in color. It should be noted that before machining the coupons, the surface of the composites was checked in order to take the ones without any visual damage (surface damage). In Figure 10, it can be seen how in both systems, the UV resistance improves when lignin is incorporated into the gel-coat, which may be due to the ability of lignin to absorb and withstand the heat produced by UV light as shown in Section 3.1 by the TGA, where the samples were subjected to high temperatures.

Analyzing the results obtained, it can be seen that formulations containing Organsolv suffer less damage. That is, the higher the amount of Organsolv, the less the color change. This might be because of the quality of this lignin, as noted in the mechanical properties section.

On the other hand, with regard to the formulations with Kraft lignin, they show better results than the reference one. However, this lignin confers lower UV resistance than Organsolv lignin.

In order to avoid a margin of error in the results, two samples of each formulation were studied, as a higher number meant higher lignin consumption. This is a high cost in quantities destined for the laboratory.

**Figure 11:**
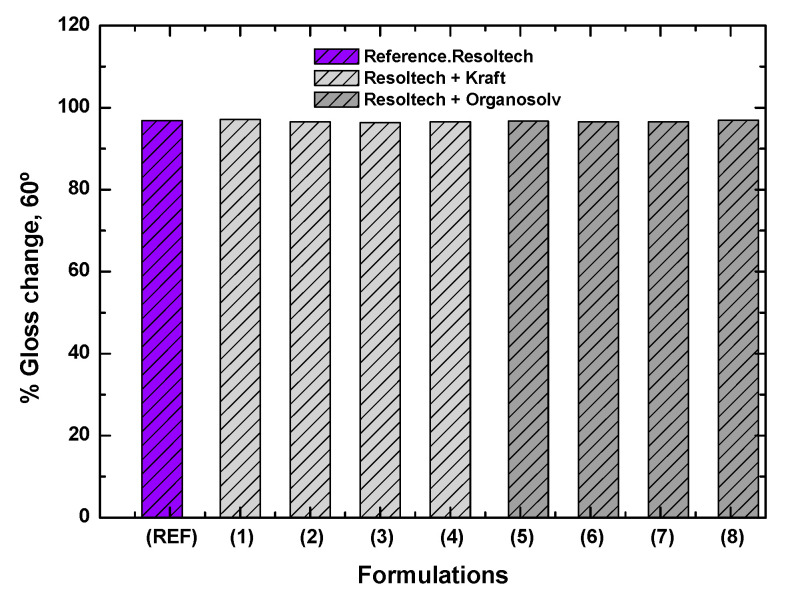
Gloss/brightness change in the developed composites.

As it is of importance that the gel-coat has a good finish, the gloss of all of the test specimens was also measured before and after the QUV aging process. The gloss was measured at three different angles (20°, 60°, and 80°), but only the values of the 60° angle were taken into account to represent the results and the percentage change. Figure 11 shows that in both systems, the percentage change is very similar; so, it can be said that the lignin does not influence the change in gloss. All of the gloss changes in the 60° arrangement are around 96%, which indicates that it is a matte surface and that there is no relevant change in gloss.

### 3.5. Fire Behavior

**Figure 12:**
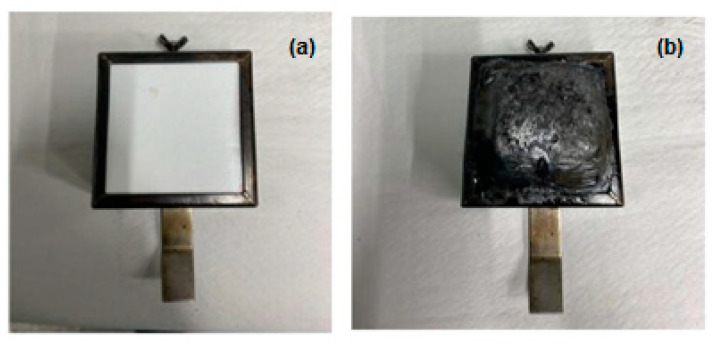
Composite samples (**a**) before and (**b**) after fire exposure.

This section shows the results obtained in the fire tests of the specimens with the calorimetric cone with radiation of 50 kW/m^2^ and a distance of 60 mm. It should be noted that these specimens have not been exposed to UV aging. Figure 12 shows the before and after of one of the specimens when subjected to this test.

One of the most important parameters to be taken into account (as mentioned in Section 2.2) in this test is the MARHE; among others, this value is used for screening and certifying different formulations of materials in the railway sector. The lower the MARHE value, the higher the fire resistance of the material. Table 4 shows the results of fire characterization.

**Figure 13:**
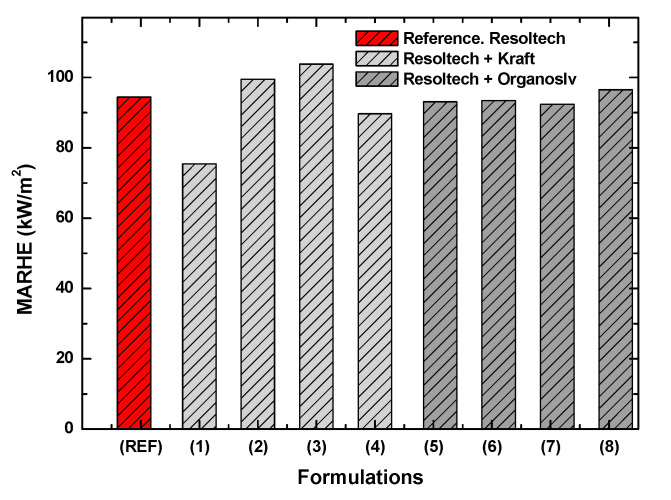
MARHE results obtained after cone calorimeter test.

As can be seen in this table, the results obtained are quite promising, since the MARHE is around 90 kW/m^2^, which makes the material suitable for closed environments. If the value obtained in the reference formulation is compared with those in which lignins are used, it can be seen that formulation 1, which contains 1%wt Kraft lignin, considerably improves the fire resistance. However, if an excess of this lignin is added (formulations 2 and 3), this effect is reversed. Formulation 4, which contains 5%wt lignin, is similar to the reference, so it is not considered to present improvements when looking at the results obtained with 2.5 and 3.5%wt lignin. On the other hand, the formulations with Organosolv present very similar values to the reference. In other words, analyzing the data in detail, it can be concluded that the most promising system, together with the Resoltech system, is the one that includes 1%wt lignin in its formulation. In Figure 13 shown below, the MARHE results can be seen in a clearer way:

To summarize, looking at all of the results obtained, it was found that lignin significantly improves the UV resistance of the composite without greatly affecting other properties, such as flexural strength. In fact, in the case of organosolv lignin, there was an improvement in these properties. In addition, thanks to the ability of lignin to withstand high temperatures, it also improved the fire resistance of the composites manufactured.

## 4. Conclusions

After analyzing the results obtained, the following conclusions were reached:

- In the DSC tests, when analyzing the lignin in the gel-coat, it was observed that the Organosolv lignin minimally alters the curing curve, and in the case of the formulations with different amounts of of Kraft lignin, do not show significant differences compared to the reference.
- Thermogravimetric analysis has shown that the gel-coat is resistant to high temperatures, as it does not degrade up to 287 °C. With respect to the lignins analyzed, Kraft lignin has a higher degradation temperature than Organosolv. No inhibition of the curing curve with lignins was observed.
- After analyzing the rheological behavior, it was found that the Resoltech system is suitable for processing in-vacuum resin infusion technology.
- With regard to the mechanical properties, it was observed that formulations 6 and 7 (formulations with organosolv lignin) show an improvement of around 20% with respect to the reference (120 MPa) in terms of strength, reaching 140–150 MPa. In relation to the modulus, the maximum modulus reached is 11,000 MPa, which is presented by the reference formulation. It should be noted that this value is also reached in formulations 1 (1%wt Kraft) and 7 (3.5%wt organosolv).
- Analyzing the color change, it was observed that the addition of lignin in the gel-coat improves the properties against UV attack. This improvement was observed, to a great extent, in those formulations with Organosolv lignin. However, it should be noted that certain improvements were also observed with Kraft lignin. As far as gloss is concerned, the addition of lignin has no effect since the surface is matte, and QUV aging did not alter the gloss of the composites.
- It was also observed that some of the formulations have MARHE values less than 90 kW/m^2^, which allows us to conclude that these composites could be used inside and outside long-distance trains. In other words, these composites comply with the HL2 (hazard level 2) classification. With regard to lignins, Kraft lignin confers better fire properties than Organosolv lignin.

Considering that the main focus of this work was UV resistance, the optimal formulation is formulation 7 (Resoltech system with 3.5%wt organosolv). It should be noted that, analyzing all of the results, the materials made with this formulation can be applied to the exterior walls (R7 according to EN45545-2:2020 classification) and end walls (R17 according to EN45545-2:2020 classification) of trams.

## Figures and Tables

**Table 1:** Gel-coat formulations used for the manufacturing of sustainable composites.

Formulation	Kraft Lignin (%)	Organosolv Lignin (%)
Reference	-	-
1	1.0	-
2	2.5	-
3	3.5	-
4	5.0	-
5	-	1.0
6	-	2.5
7	-	3.5
8	-	5.0

**Table 2:** DSC results (enthalpy and maximum exotherm temperature) for the system, Resoltech 1800Eco.

	ΔH (J/g)	Tc (°C)
Resoltech 1800eco system	450	117

**Table 3:** Theoretical enthalpy results of the developed formulations.

Formulation	ΔH Experimental (J/g)	% Gel-Coat	ΔH Theoretical (J/g)	Tc (°C)
Reference	224.90	100	224.90	98.96
1	193.74	99.19	223.08	99.96
2	186.85	98.07	220.56	99.45
3	185.40	97.32	218.87	99.30
4	209.44	96.21	216.38	96.90
5	193.49	99.19	223.08	99.61
6	191.87	98.07	220.56	100.11
7	181.70	97.32	218.87	99.45
8	175.88	96.21	216.38	98.78

**Table 4:** Results obtained in the cone calorimeter of the biobased composites developed.

Formulation	Ignition Time (s)	Extinction Time (s)	MARHE (kW/m^2^)	Qmax (kW/m^2)^
Reference	55	>1200	94.4	247.6
1	43	>1200	75.4	157.4
2	37	1159	99.5	247.4
3	37	1147	103.8	375.5
4	47	>1200	89.7	241.0
5	45	>1200	93.1	211.1
6	61	>1200	93.4	201.1
7	53	>1200	92.4	239.1
8	39	>1200	96.5	263.3

## Data Availability

The data are available in the manuscript.
